# Pathology of Bovine Tuberculosis in Three Breeds of Dairy Cattle and Spoligotyping of the Causative Mycobacteria in Ethiopia

**DOI:** 10.3389/fvets.2021.715598

**Published:** 2021-09-21

**Authors:** Mulualem Ambaw, Benti Deresa Gelalcha, Berecha Bayissa, Adane Worku, Aster Yohannis, Aboma Zewude, Gobena Ameni

**Affiliations:** ^1^Ethiopian Institute of Agricultural Research, Kulumsa Agricultural Research Center, Assela, Ethiopia; ^2^School of Veterinary Medicine, College of Agriculture and Veterinary Medicine, Jimma University, Jimma, Ethiopia; ^3^Vaccine Production and Drug Formulation Directorate, National Veterinary Institute, Bishoftu, Ethiopia; ^4^Aklilu Lemma Institute of Pathobiology, Addis Ababa University, Addis Ababa, Ethiopia; ^5^Ethiopian Institutes of Agricultural Research, Holeta Agricultural Research Center, Holeta, Ethiopia; ^6^Malaria and Neglected Tropical Diseases, Ethiopian Public Health Institute, Addis Ababa, Ethiopia; ^7^Department of Veterinary Medicine, College of Agriculture and Veterinary Medicine, United Arab Emirates University, Al Ain, United Arab Emirates

**Keywords:** bovine tuberculosis, cattle breed, molecular typing, pathology, Ethiopia

## Abstract

Different breeds of cattle were observed to have a variable degree of susceptibility to bovine tuberculosis (bTB). The screening of bTB was conducted on 720 dairy cattle consisting of three breeds using the single intradermal cervical comparative tuberculin (SICCT) test. Besides this, 43 SICCT test-positive cattle were used to compare the severity of the pathology of bTB among the three breeds and to identify the causative mycobacteria using spoligotyping. The overall SICCT test positivity was 17.92% (129/720) by pooling all animals in the three farms. There was a significant difference in SICCT test positivity among the three breeds (χ^2^ = 71.06; *p* < 0.001); the highest (25.34%) was recorded in the crossbreed followed by the Boran breed (10.08%), while the least (3.14%) was recorded in the Jersey breed. On other hand, the highest median pathology score (10.0, interquartile range, IQR = 6.0–17.0) was recorded in Boran followed by cross (5.0, IQR = 3.5–7.5), while the least (3.0, IQR = 2.25–3.0) was recorded in Jersey. Thus, the difference in the median pathology scores was significant [Kruskal Wallis $\chi_{\left(2\right)}^{2}$ = 18.78, *p* < 0.001] among the three breeds. Furthermore, multivariate analysis using ordinal logistic regression by considering age, sex, breed, reproductive status, and location of the farms also showed a significant [$\chi_{\left(2\right)}^{2}$ = 11.97, *p* < 0.01] difference in pathology scores among the three breeds of cattle. Even at a single-herd level at Holeta, the difference in severity of pathology between the Boran and crossbreeds was significant (*U* = 33.5; *p* < 0.01). Culture positivity was 39% in 108 suspicious tissues. Fourteen *Mycobacterium bovis* (*M. bovis*) and two *Mycobacterium tuberculosis* (*M. tuberculosis*) were isolated from the lesions. All the 14 *M. bovis* isolates belonged to SB0912, while the two *M. tuberculosis* belonged to SIT54. In conclusion, although the frequency of the SICCT test positivity was high in the crossbreed, a more severe pathology was observed on the Boran (zebu) breed. In addition *M*. *tuberculosis* was isolated from TB lesions of dairy cattle, demonstrating the role of *M. tuberculosis* in causing TB in cattle.

## Introduction

Bovine tuberculosis (bTB) is a chronic infectious disease of cattle and is characterized by the formation of tubercles in the lungs, lymph nodes, intestine, kidney, and other tissues (1). bTB is primarily caused by *Mycobacterium bovis* (*M. bovis*), although the other members of *Mycobacterium tuberculosis* complex (MTBC) have also been reported to cause bTB (2). *M. bovis* is most frequently isolated from cattle and less frequently from several other animals, including humans (2, 3). On the other hand *M*. *tuberculosis* is primarily adapted to human beings, though it is occasionally isolated from other mammals (2). Both *M. bovis* and *M. tuberculosis* belong to MTBC, which consist of mycobacterial species exhibiting a 99.9% sequence similarity with conserved 16SrRNA, with the exception of *M. canetti* (4), and are capable of causing a serious disease with a similar pathology (5). The members of MTBC known so far include *M. tuberculosis, Mycobacterium canettii, Mycobacterium africanum* subtypes I and II, *Mycobacterium bovis, Mycobacterium caprae, Mycobacterium microti, Mycobacterium pinnipedii*, the attenuated *M. bovis* Bacillus Calmette–Guerin (BCG*)* vaccine strain (6), *Mycobacterium orygis* (7), *Mycobacterium mungi* (8), *Dassie bacillus* (9), and *Chimpanzee bacillus* (10).

bTB is transmitted between animals primarily by inhalation in housed animals and through ingestion in animals grazing on a pasture contaminated with M. bovis. bTB causes a significant economic loss because of reduction in productivity, movement restrictions, screening costs, culling of affected animals, and trade restrictions (1). In addition, the disease is transmitted to humans, causing zoonotic TB in about 15% of the human population in developing countries (11).

Ethiopia is known to be the leading livestock-producing country in Africa and standing 10th in the world with its 59.5 million heads of cattle (12). Regarding breed composition, 98.20% of the total cattle in the country are zebu, while the remaining 1.8% was crossbreed and Holstein Friesian breeds (12). However, the huge livestock potential and the benefit that the country is getting from the livestock sector are not comparable because of the poor genetic potential of the zebu breed and the poor management and high prevalence of cattle diseases (13)—for example, the annual milk yield in the Boran zebu breed was observed to be 673 kg in Boran (zebu) breed as compared to 1,752 kg in the crossbreed of Holstein Friesian–Boran and to 2,678 kg in Holstein Friesian under Ethiopian conditions (14). Regarding the prevalence of diseases, 10 most important priority diseases have been identified based on their incidence and then ranked according to their impacts on (1) the livelihood of households, (2) markets and value chains, and (3) intensification pathways in the production system [(15); Table 1]. As indicated in Table 1, TB, brucellosis, echinococcosis, surra, and salmonellosis in species other than poultry do not have a control method, while all of the viral diseases and mycoplasma diseases are controlled by vaccination.

**Table 1:** The 10 top ranked livestock diseases in Ethiopia in descending order based on the degree of impact on the livelihood of households, market/value chains, and intensification of animal production (15) and their control methods.

**Rank**	**Disease ranking based on the impact on the livelihood of households**	**Disease ranking based on the impact on market and value chain**	**Disease ranking based on the impact on the intensification of livestock production**	**Availability and type of control method**
1st	Foot and mouth disease, FMD	FMD	Brucellosis	No control method
2nd	Contagious bovine pleuropneumonia, CBPP	LSD	PPR and FMD	Vaccination for both
3rd	Lumpy skin disease, LSD	Brucellosis	TB and Newcastle	TB: no control method Newcastle: vaccination
4th	Tuberculosis, TB	CCPP and Newcastle	CBPP	Vaccination
5th	Brucellosis	TB	LSD	Vaccination
6th	Contagious caprine pleuropneumonia, CCPP	Chicken pox	Gumboro	Vaccination
7th	Peste des Petits Ruminants, PPR	SGP	Salmonella	Vaccination only poultry
8th	Sheep and goat pox, SGP	Gumboro	SGP	Vaccination
9th	Newcastle disease	PPR	Chicken pox	Vaccination
10th	Surra	Echinococcosis	Surra	No control method

Regarding the estimation of the national prevalence of bTB in Ethiopia, a systematic review conducted on 56 articles demonstrated a pooled prevalence estimate of 5.8% (16), and according to this review, the prevalence of bTB was significantly (*p* < 0.001) higher in Holstein Friesian (21.6%) than in zebu (4.1%). In addition, the same review reported a higher prevalence (16.6%) of bTB in cattle kept under intensive and semi-intensive production systems than those kept in an extensive livestock production system (4.6%).

The government of Ethiopia has given attention to improve cattle production and productivity, including improving milk production, by raising the genetic potential of the zebu breed through crossbreeding with exotic dairy breeds using artificial insemination and synchronization in the dairy sheds and peri-urban areas of the country (15). The Boran breed (zebu) is the preferred zebu breed and is being used for crossbreeding with exotic breeds (mainly Holstein Friesian) by artificial insemination because of its better fitness in terms of size and productivity. The crossbreeding plan for the improvement of the productivity of dairy cattle should be integrated with the disease control strategy. bTB is endemic to Ethiopia and is one of the priority diseases of dairy cattle in the country that requires an appropriate control strategy. To this effect, generation of data on the comparative susceptibility of the different breeds of cattle to bTB is useful for identifying the relatively resistant and moderately productive breed. A few comparative studies conducted on the prevalence of bTB in Holstein Friesian, crossbreed of Holstein Friesian and zebu breeds, and zebu breed indicated that Holstein Friesian is the most susceptible breed as compared to either crossbreed or zebu breed, while the degree of susceptibility of a crossbreed was observed to be lower than of Holstein Friesian but higher than of the zebu breed (17–19). Although the reason behind the relative resistance of zebu to bTB has not been well-established, the innate immune responses, such as that of interleukin-6, could contribute a significant role in containing and clearing an infection with *M. bovis* (19). These studies were conducted in traditional farms in the three breeds that were kept on a grazing pasture. Similar studies are needed in dairy cattle that are kept in intensive and semi-intensive farms under modern dairy farming as the prevalence of bTB is influenced by cattle husbandry (18). Therefore, this study was conducted on three herds for screening 720 dairy cattle for bTB using the single intradermal cervical comparative tuberculin (SICCT) test, after which 43 SICCT test-positive dairy cattle were further recruited for slaughter and used for comparing the severity of pathology of bTB in the three breeds and identification of its causative mycobacteria using spoligotyping.

## Materials and Methods

### Study Setting and Sampling of Study Animals

The study was conducted at three state dairy farms (Adaberga, Bishoftu, and Holeta) that are located in central Ethiopia at about 40 km in the west (Adaberga and Holeta) and east (Bishoftu) of Addis Ababa, the capital of Ethiopia. These farms are owned by the Ethiopian Institute of Agricultural Research and used for the genetic improvement of Ethiopian cattle breed. The dairy cattle management system is semi-intensive, where the animals are kept in the barns, feed on concentrate, and are watered and also allowed for open grazing in the field. This husbandry system was similar across the three farms, and management is done by the same management body centrally. Prior to the study on the comparative pathology in the three breeds, the three farms were screened for bTB using SICCT test. The SICCT test was conducted using bovine and avian purified protein derivatives (PPD-B and PPD-A) obtained from Thermo-Fisher (Prionics, Lelystad, the Netherlands). Briefly, 0.1 ml PPD-A (2,500 IU/ml) was administered intradermally on the left side of the middle of the neck on the cranial side, and the same volume of PPD-B (3,000 IU/ml) was injected at a site 12 cm distant from the PPD-A site in the shoulder direction. The skin thicknesses were measured just before injection and at 72 h post-injection by the same operator using the same digital caliper, and the results were presented as a change in skin thickness (mm) between the two readings. The interpretation of the result was according to the recommendation of the World Organization for Animal Health (20). The differences in the increase of skin thickness at the bovine and avian PPD injection sites were determined. An animal was considered to be positive when the increase in skin thickness at the bovine PPD site was >4 mm more than the increase in skin thickness at the site of the avian injection. If the differential increase between the two sites was equal to 1 mm or between 1 and 4 mm, the animal was considered negative or inconclusive, respectively.

The total number of animals in the three herds was 720. The farm at Adaberga consisted of 244 animals, which were comprised of crosses and Jersey breeds. The herd size of the farm at Bishoftu was 114, all of which were crossbreed, while there were 362 animals in the farm at Holeta and they were comprised of Boran zebu and crossbreeds. After screening the 720 animals with the SICCT test, 43 positive cattle were selected for slaughter from among the 129 SICCT test-positive cattle identified from the three farms. Thus, 12 Boran (zebu), 26 Holstein Friesian × Boran cross, and five Jersey breeds were used for studying the comparative pathology.

The 43 strong positive animals were transported by trucks under strict safety to the Addis Ababa City Abattoir and humanly killed using knife stunning. The butchers and the meat inspectors used a personal protective equipment while slaughtering and during the postmortem examination. Tissues with lesions were collected into universal bottles in 0.9% saline and transported to the TB Laboratory at the Aklilu Lemma Institute of Pathobiology, Addis Ababa University for culturing. The remaining tissues and carcasses with TB were incinerated and disposed. The tissues were processed for isolation of mycobacteria in Biosafety level III laboratory. The leftover infected samples were disposed after autoclaving at 121°C for 15 min.

### Postmortem Examination and Pathology Scoring

The reactors were euthanized humanely and examined for gross lesion of bTB in detail by removing the lungs and lymph nodes (LNs). All the seven lobes of the lungs were inspected at the surface and palpated for the presence of TB lesions. Each lobe was then sectioned into slices to facilitate the detection of TB visible lesions (VLs). Similarly, LNs, including the left and right parotid, left and right mandibular, left and right lateral retropharyngeal, left and right medial retropharyngeal, cranial and caudal mediastinal, left and right bronchial, hepatic, and mesenteric LNs, as well as left and right tonsils, were sliced into thin sections and inspected for the presence of VLs. The severity of gross lesions was scored by a semi-quantitative scoring procedure as previously described by Vordermeier et al. (21). Briefly, the lesions in the lobes of the lungs were scored separately as follows: 0, no visible lesions; 1, no gross lesions but lesions apparent on slicing of the lobe; 2, fewer than five gross lesions; 3, more than five gross lesions; and 4, gross coalescing lesions. The scores of the individual lobes were added up to calculate the lung score. Similarly, the severity of gross lesions in individual LN was scored as follows: 0, no gross lesion; 1, a small lesion at one focus (just starting); 2, small lesions at more than one focus; and 3, extensive necrosis. Individual LN scores were added up to calculate the total LN score for each LN/tissue category. Finally, the LN and lung pathology scores were added together to give the total pathology score per animal.

### Specimen Collection and Isolation of Mycobacteria

The standard operating procedure described by the World Organization for Animal Health (20) was used for the culturing of tissue lesions. Suspicious TB lesions were collected from 41 dairy cattle for mycobacterial isolation. A total of 108 suspected tuberculous lesions were aseptically collected into sterile universal bottles in 0.9% saline solution and transported to the laboratory in a cold chain. The specimens were sectioned into pieces with sterile blades, minced with scissors, and homogenized with sterile pestle and mortar. The homogenates were decontaminated by adding an equal volume of 4% NaOH and kept for 15 min and then neutralized with 1% (0.1 N) HCl (phenol red was used as an indicator). Then, the specimen was centrifuged at 3,000 rpg for 15 min, and the supernatant was discarded; finally, two drops of suspension from each sample were spread onto a slant of Löwenstein–Jensen (LJ) medium. Duplicates of LJ were used; one was enriched with sodium pyruvate, while the other was enriched with glycerol. The cultures were incubated aerobically at 37°C for about 8 weeks with weekly observation for the growth of colonies.

### Spoligotyping

Spoligotyping was conducted following the protocol developed by Kamerbeek et al. (22), which involves amplification, hybridization, and detection steps. The colonies were heated at 85°C in a water bath for 1 h, and the released DNA was used as a template to amplify the direct repeat (DR) region of the *M. tuberculosis* complex by polymerase chain reaction (PCR) using oligonucleotide biotin-labeled primers derived from the DR sequence, RDa (5′GGTTTTGGGTTTGAACGAC3′), and RDb (5′CCGAGAGGGGACG GAAAC3′) (22).

A total volume of 25-μl reaction mixtures that consisted of 12.5 μl of HotStarTaq Master Mix (Qiagen), 2 μl of each primer (20 pmol each), 5-μl suspension of heat-killed cells, and 3.5 μl distilled water was used for running the PCR. The mixture was heated for 15 min at 96°C and then subjected to 30 cycles of 1-min denaturation at 96°C, annealing at 55°C for 1 min, and extension at 72°C for 30 s. Then, final stabilization was done at 72°C for 10 min. Thereafter, the PCR product was denatured using a thermocyler at 96°C for 10 min and then kept on ice so as to prevent the renaturing of the PCR products. The denatured PCR product was loaded onto a membrane covalently bonded with a set of 43 spacer oligonucleotides, each corresponding to one of the unique spacer DNA sequences within the DR locus of the *M. tuberculosis* complex, and then hybridized at 60°C for 1 h. After hybridization, the membrane was washed twice for 10 min in 2 × SSPE (1 × SSPE is 0.18 M NaCl, 10 mM NaH_2_PO_4_, and 1 mM EDTA, pH 7.7)]−0.5% sodium dodecyl sulfate (SDS) at 60°C and then incubated in 1:4,000 diluted streptavidin peroxidase (Boehringer) for 1 h at 42°C. The membrane was washed twice for 10 min in 2 × SSPE−0.5% SDS at 42°C and rinsed with 2 × SSPE for 5 min at room temperature. The hybridizing DNA was detected by the enhanced chemiluminescence (ECL) method (Amersham, Biosciences, Amersham, UK) and exposure to an X-ray film (Hyperfilm ECL, Amersham). A mixture of 10 ml of ECL reagent 1 and 10 ml of ECL reagent 2 was prepared and then added onto the membrane, and the membrane was rinsed in the solution for 5 min at room temperature. Then, the membrane was attached onto a film in the dark room, placed in the cassette, and then incubated for 15 min at room temperature. The film was removed and placed in a developer solution for 2 min and then removed from the developer, rinsed with tap water for 15 s, and then placed in a fixer solution for 1 min. Finally, the film was dried and used for the interpretation of the result. The presence of the spacer was identified as a black square, while the absence of the spacer was identified as a white square on the film. The black squares were converted to 1, while the white squares were converted to 0 and then transferred to the Mycobacterium bovis Spoligotype Database for the identification *M. bovis* strains. Furthermore, the identification of strains of *M. tuberculosis* was done 1 and 0 into the spoligotype international type (SIT)–VNTR international type database.

### Data Management and Analysis

Statistical Package for Social Sciences (SPSS) version 21 software was used for the analysis of the SICCT test data and part of the pathology data. Descriptive statistics was used for the analysis of the SICCT test data of the three herds. Univariate analysis was used to assess the associations of the risk factors with the SICCT test result, and chi-square (χ^2^) test was used for the estimation of the significance of the association. In addition, ordinal regression analysis was used for the determination of the association of explanatory factors (breed, age, sex, reproductive status, and farm location) with the severity of pathology. The fitness of this model was diagnosed using−2 log likelihood model, and the result of the diagnosis demonstrated the fitness of the final model with independent variables [$\chi_{\left(11\right)}^{2}$ = 46.56, *p* < 0.001]. Spearman correlation was to estimate the correlation between skin reaction to PPD (B-A) and the pathology score of the 43 reactors. Graph Pad Prism version 8 was used for the comparison of skin reactivity to the injection of purified protein derivatives, PPD (B-A), in the three breeds of cattle. Similarly, GraphPad Prism version 8 was used for the comparison of the severity of pathology in the three breeds. The comparison of the pathology among the three breeds was done by non-parametrical Kruskal–Wallis test for comparing the median pathology scores of the three breeds by assuming a non-pairing experimental design, while, on the other hand, non-parametric Mann–Whitney *U*-test was used for the comparison of the severity of pathology between the two breeds using the median scores.

## Results

### Reactivity of the Three Herds to the SICCT Test

The SICCT test results of the three herds used for the recruitment of SICCT test-positive animals used for the pathology study are presented in Table 2. The overall SICCT test positivity was 17.92% (129/720) when all the animals were pooled together. When individual farms are considered, the SICCT test positivity were 27.2% (31/114), 25.41% (92/362), and 2.26% (6/244) in the farms located at Bishoftu, Holeta, and Adaberga, respectively. The difference in SICCT test positivity among the three farms was significant (χ^2^ = 85.58; *p* < 0.001; Table 2). Similarly, there was a significant difference in SICCT test positivity among the three breeds (χ^2^ = 71.06; *p* < 0.001); the highest SICCT test positivity (25.34%) was recorded in the crossbreed, followed by the Boran breed (10.08%), while the least (3.14%) was recorded in the Jersey breed. Forty-three reactors, including 12 Boran (zebu), 26 Holstein Friesian × Boran crosses, and five Jersey breeds, were slaughtered and used for studying the comparative pathology among the three breeds.

**Table 2:** Association of different risk factors with the occurrence of bovine tuberculosis in three herds used for the selection of reactors for slaughtering for the investigation of pathology.

**Factors**		**No. of tested animals**	**Single intradermal cervical comparative tuberculin test result**			**Prevalence**	**χ^**2**^ test**	***P*-value**
			**Negative**	**Doubtful**	**Positive**			
Sex	Female	688	526	41	121	17.59	1.46	*p* > 0.05
	Male	32	22	2	8	25.00		
Age	<4 years	430	338	25	67	15.58	25.80	*p* < 0.001
	4–8 years	205	160	16	29	14.15		
	>8 years	85	50	2	33	38.82		
Breed	Boran	119	107	0	12	10.08	71.06	*p* < 0.001
	Cross	442	295	35	112	25.34		
	Jersey	159	146	8	5	3.14		
Body condition score	Poor	62	51	1	0	-	5.36	*p* > 0.05
	Medium	346	264	26	56	16.18		
	Good	312	233	16	63	20.19		
Reproductive status	Calf	22	22	0	0	-	^a^	
	Heifer	242	202	7	33	13.64		
	Pregnant	120	88	9	23	19.17		
	Lactating	250	185	20	45	18.00		
	Dry	65	39	5	21	32.31		
	Bull	21	12	2	7	33.33		
Location of the farm	Adaberga	244	230	8	6	2.46	85.58	*p* < 0.001
	Bishoftu	114	74	9	31	27.19		
	Holeta	362	244	26	92	25.41		
Total	720	548	43	129	17.92		

a *χ^2^ could not be computed because the data did not support the analysis*.

### Skin Reactivity to the SICCT Test and Pathology Score in the Three Breeds

**Figure 1:**
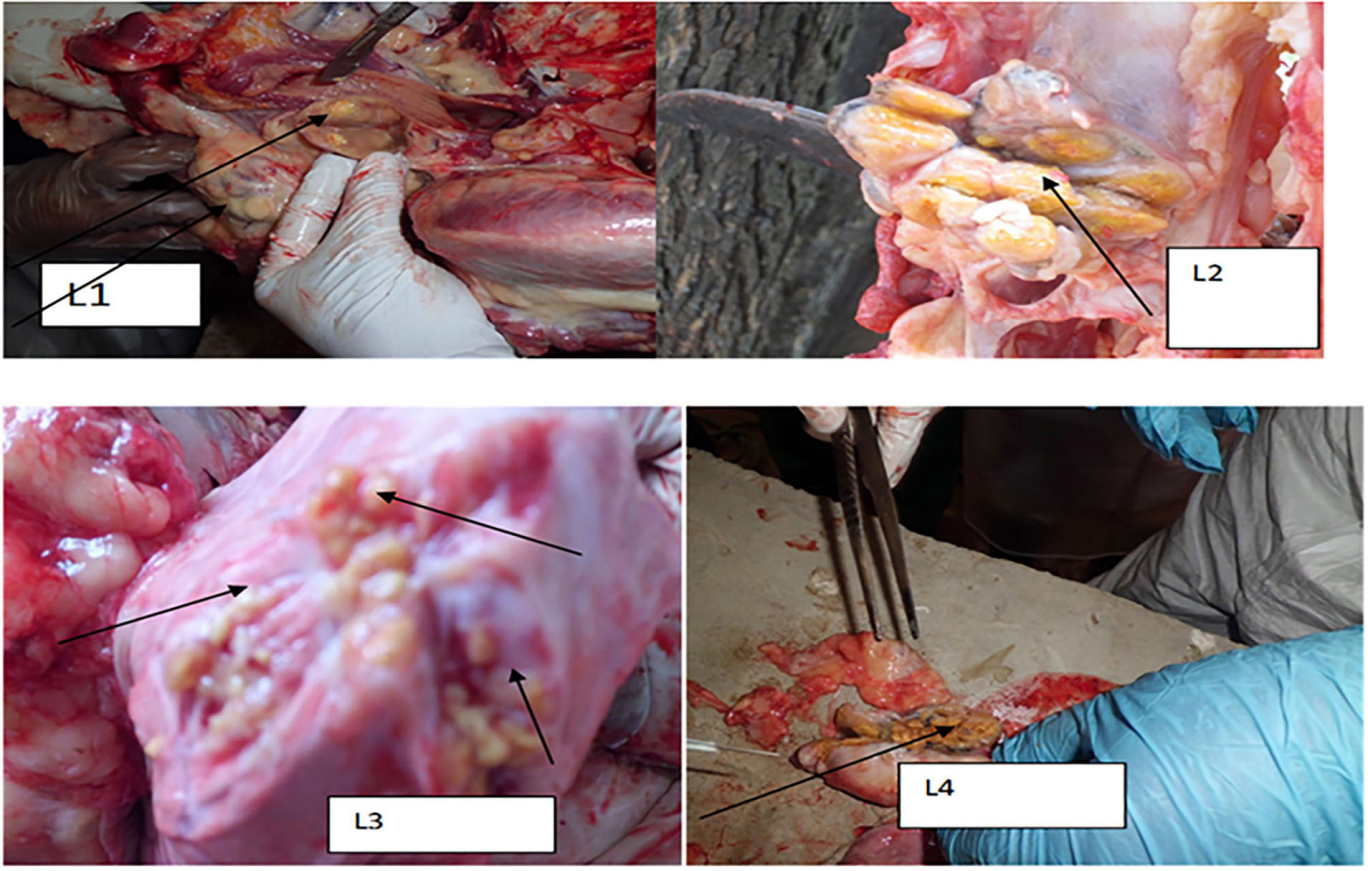
Typical TB lesions obtained in the lymph nodes and lung lobs of selected dairy cattle in central Ethiopia. L1: TB lesion on retropharyngeal lymph node; L2: disseminated TB lesions on bronchial lymph node indicated by the arrow; L3: TB lesions on the diaphragmatic lobe of the lung; and L4: calcified lesion from the mesenteric lymph node.

**Figure 2:**
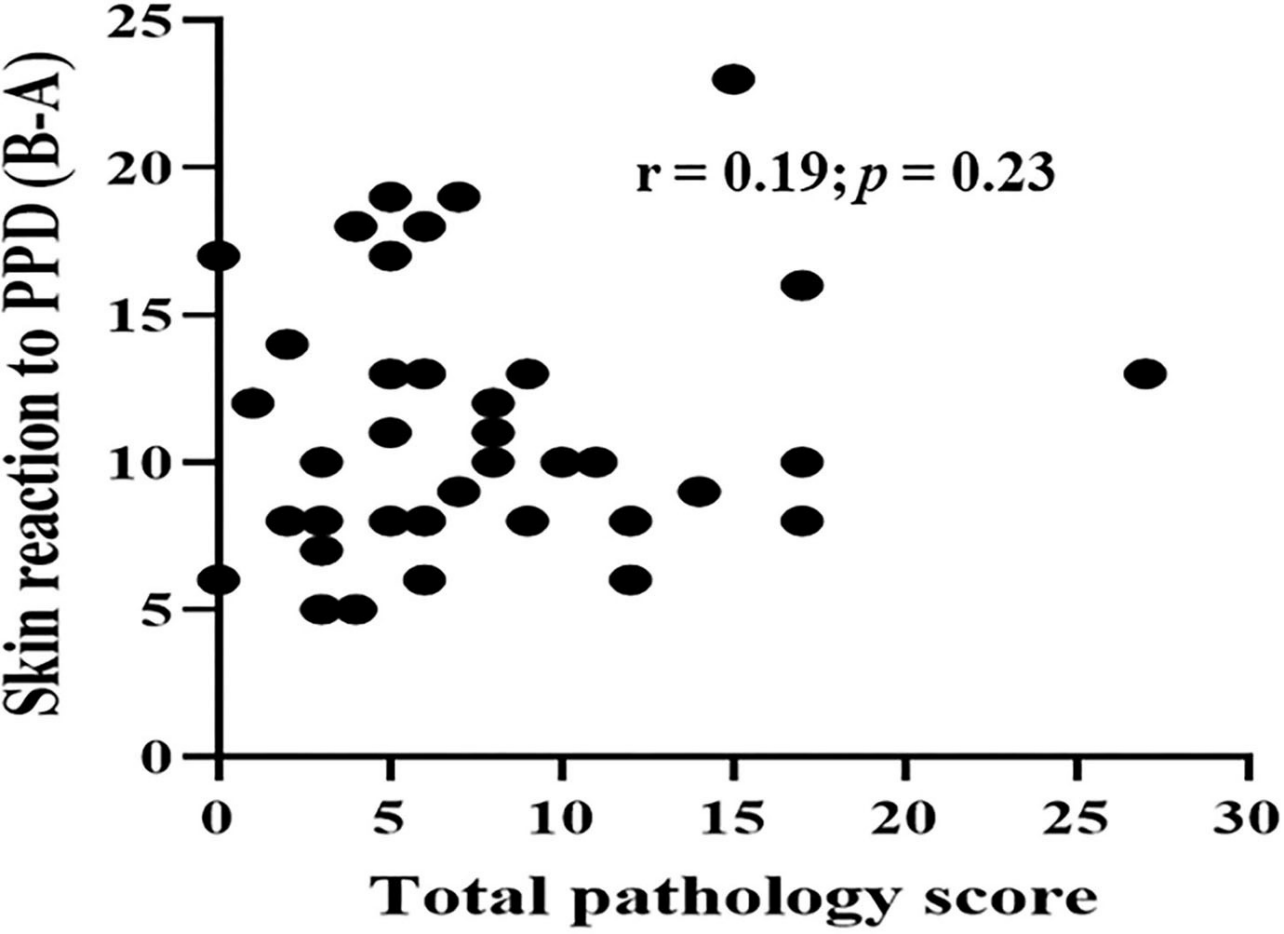
Correlation between skin reactivity and pathology score of the 43 study animals. Spearman correlation was to estimate the correlation between skin reaction to PPD (B-A) and pathology score of the 43 reactors. The correlation between two was not significant (*r* = 0.19; *p* = 0.23).

**Figure 3:**
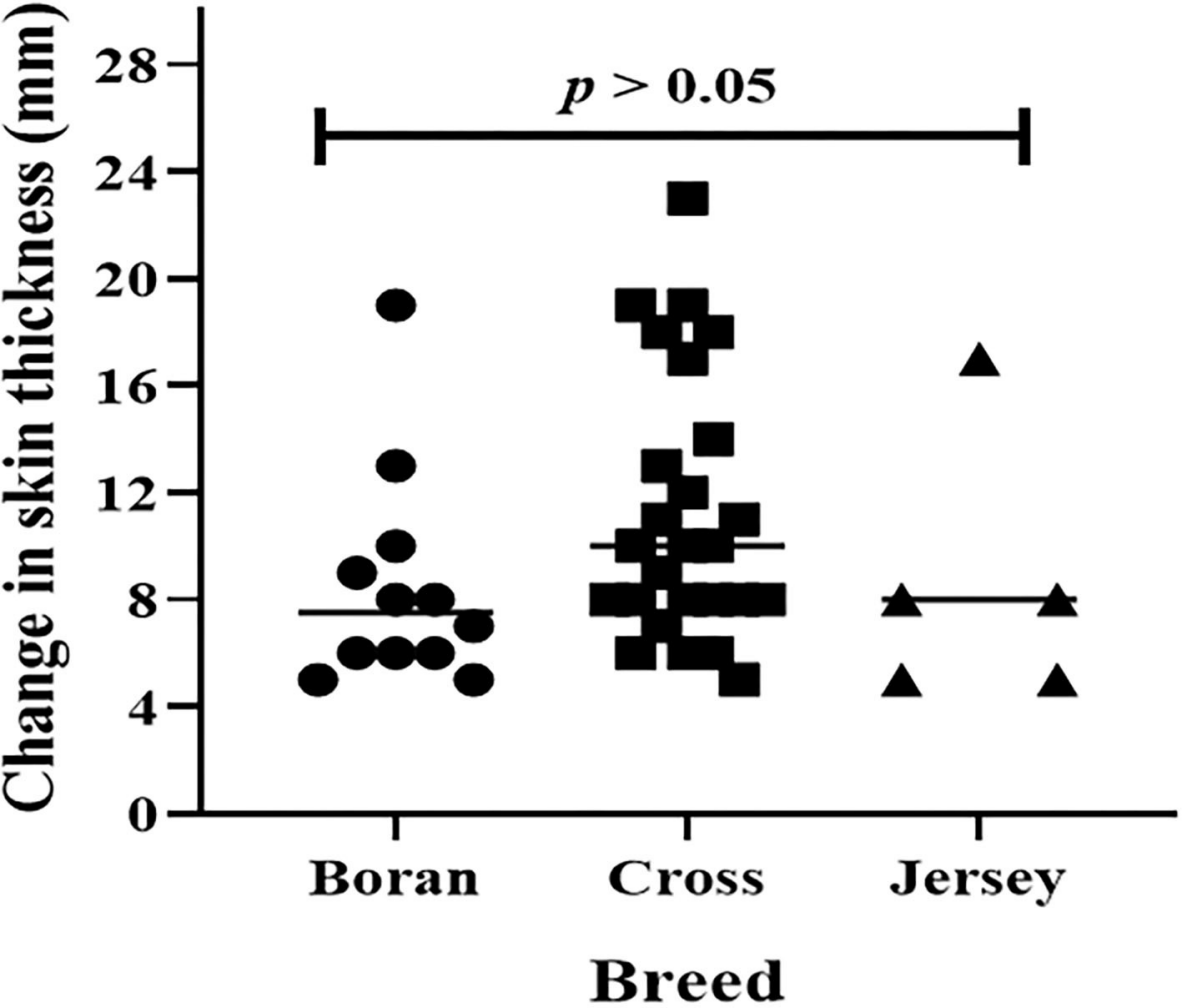
Skin reaction to purified protein derivatives, PPD (B-A) in 43 dairy animals. The horizontal lines represent a median of skin reactions in each breed. Change in skin thickness was compared among Boran, cross, and Jersey breeds using Kruskal-Wallis statistical test. There was no significant [χ^2^_(2)_ = 4.8, *p* > 0.05] difference among the breeds.

Typical TB lesions were observed in different lymph nodes (Figure 1) in 95% (41/43) of SICCT-positive cattle, but the correlation between the skin reaction to the SICCT test and the severity of pathology was not significant (*r* = 0.23; 95% CI, −0.13, 0.47, Figure 2). Change in skin thickness was compared among Boran, cross, and Jersey breeds using the Kruskal–Wallis test. The median skin thicknesses were 7.5 (interquartile range, IQR = 6.0–9.75), 10.0 (IQR = 8.0–14.75), and 8.0 (IQR = 5.0–12.5) in Boran, cross, and Jersey breeds, respectively. There was no significant [$\chi_{\left(2\right)}^{2}$ = 4.8, *p* > 0.05] difference in skin reactivity observed among the three breeds (Figure 3).

### Comparison of the Pathology of bTB in the Three Breeds of Cattle

**Figure 4:**
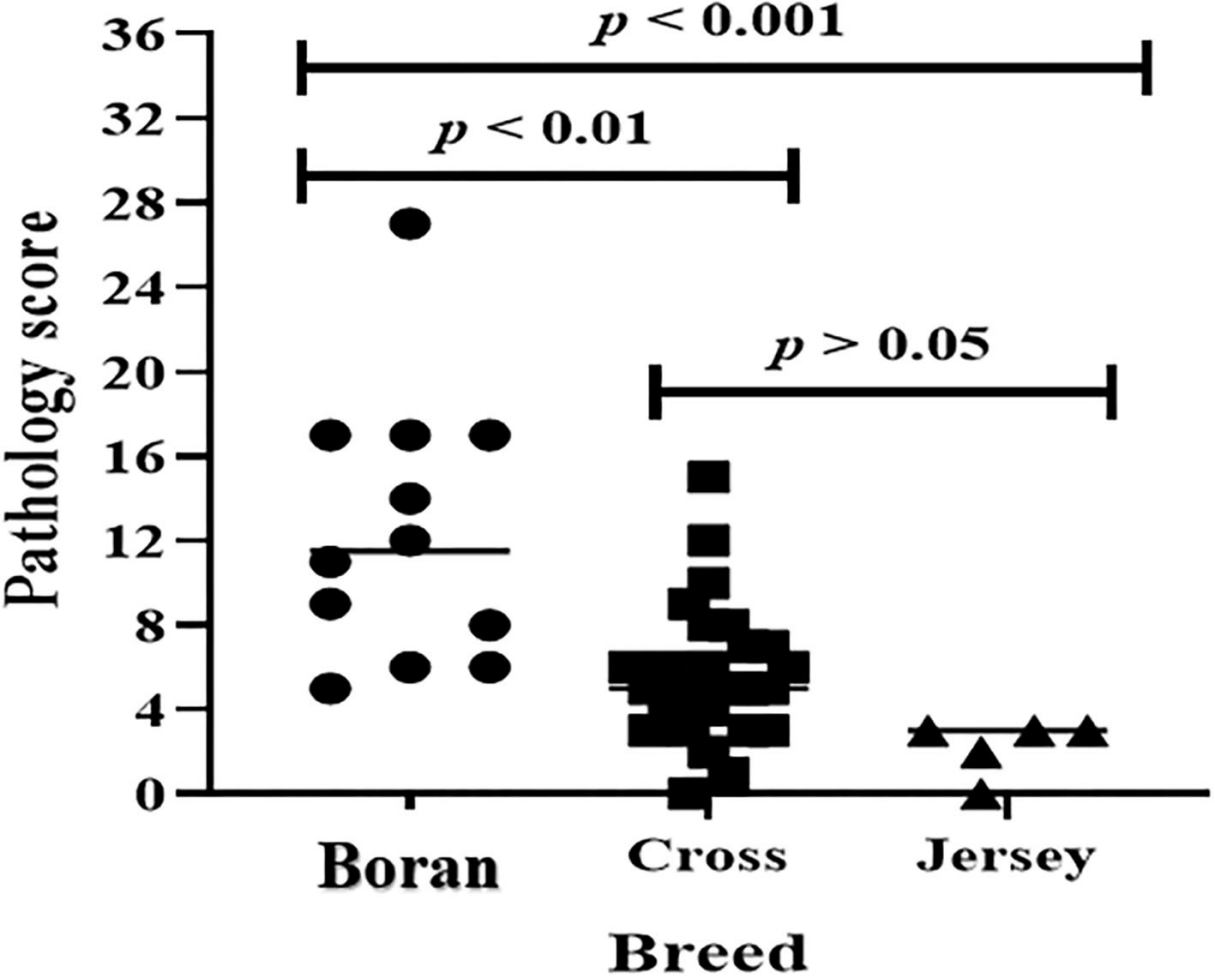
Pathology score in 43 study animals. The horizontal lines show median pathology scores of each breed. The pathology score was compared in three breeds using Kruskal-Wallis statistical test with Dunn's *post-hoc* multiple comparison test. There was significant [χ^2^_(2)_ = 18.78, *p* < 0.001] difference in pathology score among the three breeds. The difference was observed between the Boran and the cross breeds (*U* = 13.91; *p* < 0.01) as well as between the Boran and the Jersey breeds (*U* = 26.94; *p* < 0.001).

**Figure 5:**
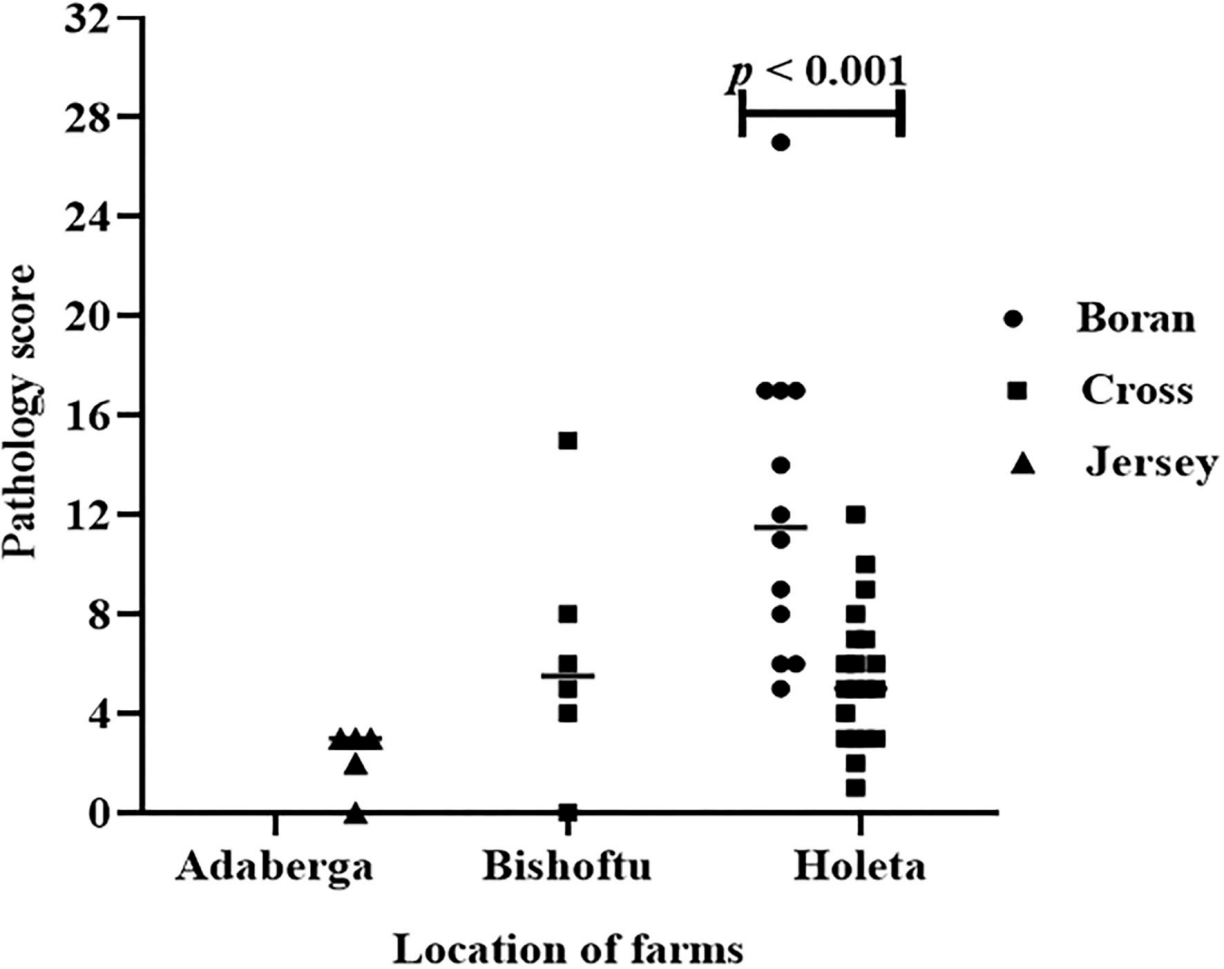
Pathology score in the three breeds in three study farms. The difference in pathology score between the Boran and cross breeds was significant (*U* = 33.5; *p* < 0.01) at farm located at Holeta. However, comparison of pathology between the breeds was not possible at the farms located at the Adaberga and Bishoftu as the positive animals in these farms belong to a single breed; Jersey at Adaberga and cross at Bishoftu.

The evaluation of the severity of pathology indicated that the median pathology scores were 10.0 (IQR = 6.0–17.0), 5.0 (IQR = 3.5–7.5), and 3.0 (IQR = 2.25–3.0) in Boran, cross, and Jersey breeds, respectively. There was a significant [$\chi_{\left(2\right)}^{2}$ = 18.78, *p* < 0.001] difference in the pathology score among the three breeds using Kruskal–Wallis statistical test with Dunn's *post-hoc* multiple-comparison test (Figure 4). The difference was observed between the Boran and the crossbreeds (*U* = 13.91; *p* < 0.01) as well as between the Boran and the Jersey breeds (*U* = 26.94; *p* < 0.001). In addition, in order to control the cofounding factors, a multivariate analysis was performed using ordinal logistic regression by considering age, sex, breed, reproductive status, and the location of the farms in the analysis. The result of this analysis still showed a significant [$\chi_{\left(2\right)}^{2}$ = 11.97, *p* < 0.01] difference in the pathology scores among the three breeds of cattle (Table 3). Thus, the difference in severity of pathology between the Boran breed and either cross (*p* < 0.05) or jersey (*p* < 0.01) breed was significant. However, no significant difference was observed in the severity of pathology between the cross and Jersey breeds (*p* > 0.05) (Figure 4). The comparison of severity of pathology was also performed in the herd located at Holeta, where two positive breeds were found, and the difference in the pathology score between the Boran and crossbreeds was significant (*U* = 33.5; *p* < 0.01) (Figure 5). However, a comparison of pathology between the breeds in the farms located at Adaberga and Bishoftu could not be performed as two or more positive breeds were not found in these farms.

**Table 3:** Association of different risk factors with the severity of pathology in 41 (two animals with 0 pathology score were not considered in the analysis) cattle using multivariable ordinal logistic regression.

**Risk factors**	**Category**	**No. of animals**	**Estimate (95% confidence interval)**	**Standard error**	**Wald χ^**2**^ test**	**df**	***P*-value**
Breed	Borana	12	4.450 (1.621, 7.279)	1.444	9.503	1	0.002
	Cross	25	2.212 (0.077, 4.346)	1.089	4.124	1	0.042
	Jersey	4	-	-		-	-
Age	≤ 4 years	22	−0.090 (−1.641, 1.456)	0.790	0.140	1	0.907
	4–8 years	7	0.935 (−1.033, 2.903)	1.004	0.867	1	0.352
	≥8 years	12	-	-		-	-
Sex	Male	2	−1.333 (−4.833, 2.167)	1.786	0.557	1	0.455
	Female	39	-	-	-	-	-
Body score	Poor	6	−0.272 (−2.216, 1.672)	0.992	0.075	1	0.784
	Moderate	17	−0.658 (−2.075, 0.759)	0.723	0.827	1	0.363
	Good	18	-	-	-	-	-
Reproductive status	Heifer	10	−0.810 (−3.250, 1.631)	1.245	0.423	1	0.516
	Pregnant	8	−2.672 (−4.759, −0.584)	1.065	6.293	1	0.012
	Lactating	11	−0.677 (−3.063, 1.709)	1.217	0.309	1	0.578
	Dry	10	-	-	-	-	-
	Bull	2	-	-	-	-	-

### Bacteriological Isolation and Identification of the Isolates by Spoligotyping

**Figure 6:**
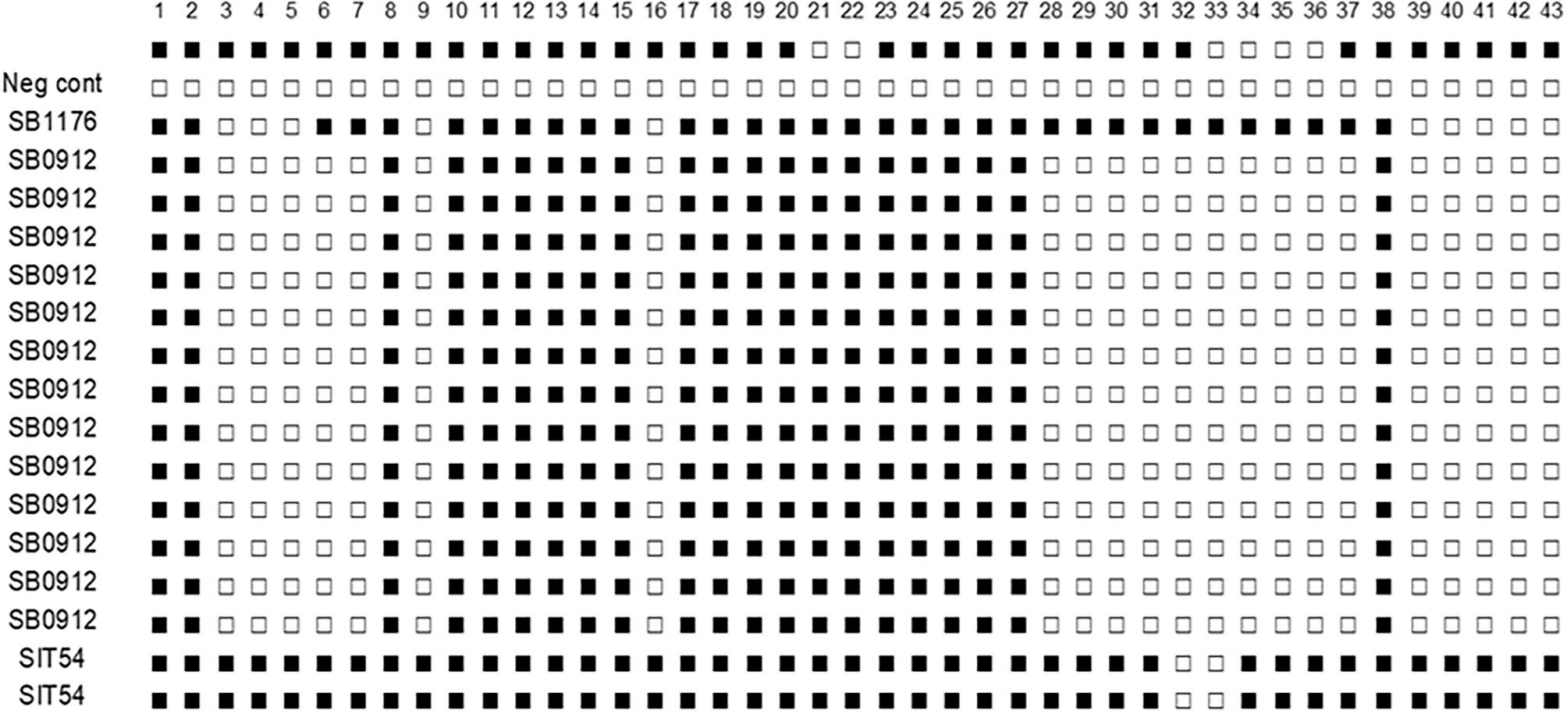
Spoligotype patterns of isolates of *M. tuberculosis* and *M. bovis* from cattle TB lesions in central Ethiopia. All of *M. bovis* isolates had the spoligotype pattern of SB0912 while the two *M. tuberculosis* had the spoligotype pattern of SIT54. SB1176 is positive control and H_2_O a negative control. The white rectangles indicate negative signals and the black rectangles represent a positive signal.

Culture positivity in the study animals was 39% (16/41) on the LJ medium. The highest (56.3%) and the lowest (2.4%) isolation rates were observed from lung lobes and mesenteric lymph nodes, respectively. From the 16 total isolates, 14 were confirmed to be *M. bovis*, and the remaining two were *M. tuberculosis* (Table 4). The 16 *M. bovis* isolates had a spoligotype pattern of B0912, while the two *M. tuberculosis* isolates had a spoligotype pattern of SIT54 (Figure 6).

**Table 4:** Distribution of tuberculosis lesion in the lymph nodes of single intradermal cervical comparative tuberculin test-positive animals and spoligotypes of *M. bovis* and *M. tuberculosis* isolated from the lesion.

**ID**	**Breed**	**Age**	**Sex**	**Farm location**	**Lesion type**	**Infection site**	**Species**	**Strain**
H211820	Cross	1 year	F	Holeta	Calcified	Right bronchial	*M. tuberculosis*	SIT54
H 20126	Cross	12 years	F	Holeta	Calcified	Left bronchial	*M. bovis*	SB0912
H 20146	Cross	12 years	F	Holeta	Spot like	Retropharyngeal	*M. bovis*	SB0912
H 25142	Cross	7 years	F	Holeta	Spot like	Mesenteric	*M. tuberculosis*	SIT54
H 21208	Boran	11 years	F	Holeta	Milliary	Caudal mediastinal	*M. bovis*	SB0912
H 21209	Boran	12 years	F	Holeta	Miliary	Retropharyngeal	*M. bovis*	SB0912
H 21206	Boran	9 years	F	Holeta	Milliary	Left bronchial	*M. bovis*	SB0912
H 29221	Boran	3 years	F	Holeta	Calcified	Right diaphragmatic lymph node	*M. bovis*	SB0912
H 21228	Boran	11 years	F	Holeta	Calcified	Right prescapular	*M. bovis*	SB0912
H 21244	Boran	11 years	F	Holeta	Calcified	Retropharyngeal	*M. bovis*	SB0912
H 26006	Boran	6 years	F	Holeta	Calcified	Retropharyngeal	*M. bovis*	SB0912
H 26006	Boran	6 years	F	Holeta	Calcified	Left bronchial	*M. bovis*	SB0912
H 25171	Cross	7 years	M	Holeta	Calcified	Mediastinal caudal	*M. bovis*	SB0912
H211146	Cross	1 year	F	Holeta	Calcified	Right bronchial	*M. bovis*	SB0912
D 102.2	Cross	4 years	F	Debrezeit	Calcified	Mammary lymph node	*M. bovis*	SB0912
D 92.5	Cross	5 years	F	Debrezeit	Calcified	Right accessory lung	*M. bovis*	SB0912

## Discussion

Screening of bTB was conducted in three farms that are located at Adaberga, Bishoftu, and Holeta in central Ethiopia. These farms belong to the Ethiopian Institute of Agricultural Research, and testing for bTB was conducted using the SICCT test for the control of the disease in these farms. A further study was conducted on 43 SICCT test-positive dairy cattle for comparing the severity of pathology of bTB in Boran zebu, crossbreed of Holstein Friesian and zebu, and Jersey breeds. In addition, mycobacteria were isolated, and strain levels were identified using mycobacterial culturing and spoligotyping.

The SICCT test positivity was compared among the three breeds, and the result indicated the highest percentage in the crossbreed, followed by the Boran breed, while the least percentage was recorded in the Jersey breed. This observation is similar with the reports of earlier studies in central Ethiopia, which reported a higher prevalence of bTB in the Holstein Friesian and the crossbreeds than in zebu breed (18, 23–26). However, those studies were based on the SICCT test and did not involve the comparison of the severity of pathology in these breeds since they were conducted in live animals. In the present study, the comparison was not only made in live animals on the basis of SICCT test but also conducted in slaughtered animals using detailed postmortem examination. The result of the SICCT test of this study agrees with the results of previous studies as indicated above.

However, the result of the detailed postmortem examination indicated a rather more severe pathology in the Boran breed than in either crossbreed or Jersey breed under a similar intensive management of cattle husbandry. This observation was disagreeing with a few studies which reported a more severe pathology in *Bos taurus* breeds such as Holstein Friesian or their crosses with zebu than in *Bos indicus* (17–19, 27). Additionally, historical comparative studies on the susceptibility of *B. taurus* and *B. indicus* in Asia and Africa indicated that *B. indicus* was resistant to bTB and also developed good protection against bTB by BCG vaccination (28–30). Carmichael (29) reported that the incidence and severity of pathological lesions of bTB were lower in zebu cattle than in Taurine breeds. However, in some of the previous studies, it was not well-elucidated if these comparative studies were conducted under similar environmental and husbandry conditions since husbandry plays a key role in the epidemiology of bTB. An intensive cattle husbandry is considered as the most important risk factor for facilitating the transmission of *M. bovis* infection [reviewed by Sihbat et al. (16) because of the close physical contact between animals, which can easily facilitate the transmission of the infection. Therefore, the comparison of the susceptibility of the two breeds to bTB should ideally be made under similar husbandry conditions so that the confounding factors can be avoided. In the present study, the three breeds were kept under a similar husbandry system, which is semi-intensive farming. In addition, the possible confounding factors such as sex, age, body condition, reproductive status, and location of the farms were considered in the analysis for controlling the effects of confounding factors. Although it is difficult to explain the reason why the pathology is more severe in zebu, keeping this breed indoors could cause stress to this breed and thereby increases the severity of TB lesions (31–33) since the zebu breed is usually kept on a pasture.

The other cause of the difference in severity of the disease could be the difference in the dates of infection, and in this case, the zebu breed had been infected earlier than the date of infection of the other two breeds. Obviously, the lesion is more severe in the late stage of infection than in the early stage of infection, and the dates of infection could be an important factor in affecting the severity of the pathology. On the other hand, no association was observed between the age of the animals and the severity of pathology as demonstrated using multiple logistic regression analysis.

The other possible reason for such difference in the severity of the lesion in these breeds could be attributed to the small number of study animals used for comparison. Additionally, the number of study animals in the three breeds was not similar which could have affected the result of the analysis. Therefore, a further study should be conducted on the comparative susceptibility of Boran breed (zebu) and Holstein–zebu crossbreed under a similar intensive husbandry system through the exposure of naïve TB-free cattle of the three different breeds of infected animals at the same time and then investigating the comparative susceptibility of the three breeds to bTB.

The proportion of gross lesion detected in SICCT test reactors was 95.4%, and it was higher than the proportions of gross lesions reported earlier by other studies (18, 23, 34, 35). The higher portion of lesion positivity could be due to the selection of strong reactors for slaughtering and postmortem examination, although there is a correlation between reactivity to PPD and the severity of pathology. The isolation rate of the bacteria from the 41 lesion-positive animals was 39% (16/41), which was higher than those reported by other workers in cattle and camel (36–38). In this study, better culture positivity was recorded in the thoracic lymph nodes and the lung lobes, followed by the retropharyngeal lymph nodes, which could suggest that both oral and respiratory routes could be important routes of infection in these dairy farms. Similarly, other authors had also reported a higher culture positivity in the lung and the thoracic lymph nodes than in the head lymph nodes (23, 39, 40). The low isolation rate in the mesenteric lymph nodes could be because most of the lesions that were seen in the mesenteric lymph node during postmortem inspection were calcified, which reduced the number of viable bacteria, leading to a low isolation rate.

The isolation of *M. bovis* from the mammary lymph node of lactating dairy cow implies public health risks as *M. bovis* can be excreted through milk and infect raw milk consumers. The isolation of *M. tuberculosis* from cattle TB lesions can suggest reverse zoonosis, suggesting the transmission of *M. tuberculosis* from animal attendants to cattle (41, 42). In addition to these two important species of mycobacteria, earlier studies reported the isolation of non-tuberculous mycobacteria from a TB suspicious lesion in cattle (26, 43). All *M. bovis* isolated by the present study had the spoligotype pattern of SB0912, suggesting the presence of an actively ongoing infection by SB0912. SB0912 was isolated from different regions of Ethiopia by other authors (38). In addition to SB0912, two *M. tuberculosis* with the spoligotype pattern of SIT54 were isolated, which agrees with the observation of other authors who isolated *M. tuberculosis* from a TB suspicious cattle lesion (38, 43–45). SIT54 is one of the frequent spoligotypes of *M. tuberculosis* being isolated from humans in central Ethiopia (46).

## Conclusions

In conclusion, although the frequency of SICCT test positivity was high in the crossbreed, a more severe pathology was observed on the Boran (zebu) breed. In addition *M*. *tuberculosis* was isolated from TB lesions of dairy cattle, demonstrating the role of *M. tuberculosis* in causing TB in cattle.

## Data Availability Statement

The original contributions presented in the study are included in the article/Supplementary Material, further inquiries can be directed to the corresponding author/s.

## Ethics Statement

Ethical review and approval was not required for the animal study because the study was conducted on dairy farms of the Ethiopian Institute of Agriculture as part of the bTB control program and lead by the staff members of the same Institute.

## Author Contributions

MA, BG, and GA conceived and designed the experiments. MA, AW, AY, and AZ performed the field and laboratory work. MA, BG, and GA participated in the data analysis. MA drafted the manuscript, while BG and GA edited the manuscript. BB data analysis and edition of the revised copy of the manuscript. All the authors read and approved the final manuscript.

## Funding

This research project was supported by the Ethiopian Institute of Agriculture and the Addis Ababa University.

## Conflict of Interest

The authors declare that the research was conducted in the absence of any commercial or financial relationships that could be construed as a potential conflict of interest.

## Publisher's Note

All claims expressed in this article are solely those of the authors and do not necessarily represent those of their affiliated organizations, or those of the publisher, the editors and the reviewers. Any product that may be evaluated in this article, or claim that may be made by its manufacturer, is not guaranteed or endorsed by the publisher.
